# Comparative gene expression profiling analysis of urothelial carcinoma of the renal pelvis and bladder

**DOI:** 10.1186/1755-8794-3-58

**Published:** 2010-12-15

**Authors:** Zhongfa Zhang, Kyle A Furge, Ximing J Yang, Bin T Teh, Donna E Hansel

**Affiliations:** 1 Laboratory of Cancer Genetics, Van Andel Research Institute, 333 Bostwick Avenue NE, Grand Rapids, MI 49503, USA; 2 Laboratory of Computational Biology, Van Andel Research Institute, 333 Bostwick Avenue NE, Grand Rapids, MI 49503, USA; 3 Department of Pathology, Northwestern University, Feinberg School of Medicine, 251 E. Huron, Chicago, Illinois 60611, USA; 4 NCCS-VARI Translational Research Laboratory, National Cancer Centre, 11 Hospital Drive, Singapore 169610; 5 Pathology and Laboratory Medicine Institute, Glickman Urological and Kidney Institute, Genomic Medicine Institute and Taussig Cancer Institute, Cleveland Clinic, 9500 Euclid Avenue, Cleveland, OH 44195, USA; 6 Center for Systems and Computational Biology, The Wistar Institute, Philadelphia, PA 19104, USA

## Abstract

**Background:**

Urothelial carcinoma (UC) can arise at any location along the urothelial tract, including the urethra, bladder, ureter, or renal pelvis. Although tumors arising in these various locations have similar morphology, it is unclear whether the gene expression profiles are similar between the upper-tract (ureter and renal pelvis) and lower-tract (bladder and urethra) carcinomas. Because differences may facilitate different screening and treatment modalities, we sought to examine the relationship between urothelial carcinoma of the renal pelvis (rUC) and urothelial carcinoma of the bladder (bUC).

**Methods:**

Fresh tumor tissue was collected from patients with bUC (n = 10) and benign mucosa from the bladder of individuals undergoing resection for non-UC conditions (n = 7). Gene expression profiles from these samples were determined using high-throughput Affymetrix gene expression microarray chips. Bioinformatic approaches were used to compare the gene expression profiles of these samples with those of rUC samples and normal kidney samples that had been described previously.

**Results:**

Using unsupervised analytic approaches, rUC and bUC were indistinguishable. Yet when a supervised analytic approach was used, a small number of differentially expressed genes were identified; these differences were most likely limited to a single pathway--the chloride ion binding activity pathway--which was more frequently activated in rUC than in bUC.

**Conclusions:**

We found that the gene expression profiles of UCs from the upper and lower tract were extremely similar, suggesting that similar pathogenic mechanisms likely function in the development of these tumors. The differential expression of genes in the identified pathway may represent a new avenue for detection of upper-tract tumors.

## Background

Urothelial carcinoma (UC) can arise anywhere along the epithelial lining of the urinary tract, including the renal pelvis, ureter, bladder, and urethra. Traditionally, upper-tract tumors are considered to be those arising from the renal pelvis and ureter, whereas lower-tract tumors arise in the bladder and urethra. Despite similar morphologic appearances, upper- and lower-tract UCs have been proposed to represent unique entities, based on their differing locations and embryonic derivation from distinct structures [1]. In addition, exposure to toxins may be more pronounced in the bladder due to its storage function, perhaps suggesting different initiating factors in cancer development.

In general, UCs of the upper tract have been associated with a more aggressive disease course and are often not diagnosed until the more-advanced stages, relative to UCs of the bladder [2-5]. Many investigators believe that the minimal sub-epithelial connective tissue and muscularis of the upper urinary tract may predispose it to early tumor invasion [2-5]. However, upper-tract tumors are often more challenging to diagnose, as patients are often asymptomatic and urine cytology may not be as sensitive to these more distant lesions, suggesting that a delay in diagnosis may also account for more aggressive tumor behavior.

Regardless of the inciting mechanism or long-term behavior, an understanding of the differences or similarities in the genetic profiles of UCs of the upper and lower tract is critical in defining the utility of new diagnostic and treatment protocols. In order to address this, we collected samples from patients with lower-tract UC (bladder; bUC) and benign mucosa samples from bladder of individuals undergoing resection for non-UC conditions; their gene expression profiles were obtained using Affymetrix gene expression profile arrays. These profiles were compared with profiles from upper-tract UC (renal pelvis; rUC) and benign renal pelvic mucosa (rNO). To our knowledge, this is the first report to compare the gene expression profiles of UCs of the upper and lower tracts.

## Methods

The bladder samples were collected from the Cleveland Clinic Foundation which included 10 samples of muscle-invasive UC arising in the bladder (bUC), and 7 samples of benign bladder urothelium obtained from patients undergoing cystectomy for interstitial cystitis or non-functioning bladder. We compared the gene expression profiles of these tumors with those of 14 normal kidney and 14 urothelial carcinoma from renal pelvis, most of them (n = 13) were used in our previous publication [6] (with one new addition for each class in this study). Informed written consent of each patient was obtained, and this study was approved by the Institutional Review Boards of Cleveland Clinic, the Van Andel Research Institute, and Spectrum Health Hospital. All specimens were obtained within 15 minutes of surgical extraction and were immediately opened and samples snap-frozen in liquid nitrogen. All samples were reviewed by an expert urology pathologist (D.E.H.). Samples with >10% necrosis or contaminating renal parenchyma on frozen section of the specimen were excluded from evaluation. Although the tissue samples were obtained at different time periods and different locations, all followed the same standard acquisition procedure, and the microarray data were all obtained by the same facility in our laboratory. This effectively reduced the likelihood of having batch effect in the generated data set. The samples and the class abbreviations are summarized in Table 1.

**Table 1:** Summary of samples and abbreviation of classes used in the study.

CLASS	#CLASS	Details
**bNO**	7	Tissue samples from benign bladder urothelium (lower tract)

**bUC**	10	Tissue samples from patients with UC of the bladder (lower tract)

**rNO**	14	Tissue samples from benign renal pelvic mucosa (upper tract from renal pelvic) described in [6] with one additional sample.

**rUC**	14	Tissue samples from patients with UC in the upper tract (renal pelvic) described in [6], with one additional sample.

**Total**	45	

### Raw gene expression levels

Ten micrograms of total RNA was processed for the expression microarrays using the Affymetrix GeneChip one-cycle target labeling kit (Affymetrix, Santa Clara, CA) according to the manufacturer's recommended protocols. The resultant biotinylated cDNA was fragmented and then hybridized to the GeneChip human genome (54,675 probe sets in total, including more than 35,000 human genes; Affymetrix). The arrays were washed, stained, and scanned using the Affymetrix Model 450 Fluidics Station and Affymetrix Model 3000 scanner using the manufacturer's recommended protocols.

Expression values were generated by using Microarray Suite (MAS) v5.0 software (Affymetrix). The probes were redefined using updated probe set mappings (Bioc package: *hs133phsentrezgcd *[7]). The hybridizations were normalized using the robust multichip averaging (rma) algorithm implemented in Bioconductor package *affy *(http://www.bioconductor.org/, [8]) to obtain a summary expression value for each probe set of genes [9-11]. This resulted in a gene expression intensity data set containing more than 17,000 rows (genes), each of which has one numeric value representing its relative expression intensity in the sample.

### Gene expression summary

Gene expression levels were summarized according to the genes' physical locations using the regional expression biases package (*reb*) in Bioconductor [9,10]. The algorithm groups the gene expression intensities by the associated gene locations. For each region (cytoband), a general test (such as binomial or *t*-test) is applied to determine if the gene expressions in the region are collectively higher or lower than the reference expressions. The test statistics are then output for each sample and for each cytogenetic region. As large-scale genomic gain (or loss) is often accompanied by overexpressed (or underexpressed) genes within the region [12], this summary was often used as a surrogate to detect the underlying genomic alterations [13-15].

### Gene expression profiling study through unsupervised and supervised clustering algorithm

A hierarchical clustering algorithm was used for finding (unsupervised) clusters based on the Euclidean distance for dissimilarities of gene expression profiles among the samples. The slightly modified "plot.phylo" function from the analysis of phylogenetics and evolution (*ape*) package was used to display the clustering results [16]. The interquartile range (IQR) and coefficient of variation (CV) were used to filter the genes (in log scale of the intensities) for the unsupervised clustering study. The IQR was defined to be the distance between the first and third quartiles of the data vector. The CV value of a data vector, on the other hand, was defined to be the standard deviation divided by its mean value. We used IQR > 0.9 and CV > 0.1 as our filtering criteria, which resulted in a set of about 3500 genes.

To study the stability of the clustering trees, we used consensus clustering analysis[17]. Instead of building one tree from the data set, this algorithm will build as many trees as specified by bootstrap sampling the genes from gene space and then find the percentage of occurrence of a cluster among all trees. The higher this percentage is, the more stable the cluster, and the more similar are the gene expression profiles within the cluster. The Euclidean distance was used in this study. A total of 500 bootstrapped samples with replacement on the gene space were used.

We used the significance analysis of microarrays (SAM) algorithm to pick the differential genes [18]. The most informative genes for separating bladder and renal UCs were selected in two steps. In the first step, genes were filtered as before to exclude those genes with little variation across the samples. In the second step, the top genes were picked by statistical (2-sample) testing by directly comparing the gene expression intensities between samples of rUC and bUC. The p-values for genes in the filtered gene list after the first step were calculated by the Wilcoxon rank sum and signed rank tests [19]. The q-values were calculated by John Storey et al.'s procedure [20] for multiple test adjustment. The q-value is more proper for multiple testing, as it was designed to control the False Discovery Rate (FDR) when the number of tests is large. The individual test p-values are generally overestimated when there is more than one hypothesis to be tested simultaneously.

We set 0.05 as our significance level in all tests reported in this paper; it was also the default FDR level for the selection of differential genes when the SAM algorithm was used, unless otherwise explicitly specified. All calculations were implemented in the R environment at version 2.7.2 or higher [21].

## Results

### Regional gene expression bias analysis of upper- and lower-tract UCs relative to normal references for inference of underlining genomic alterations

**Figure 1:**
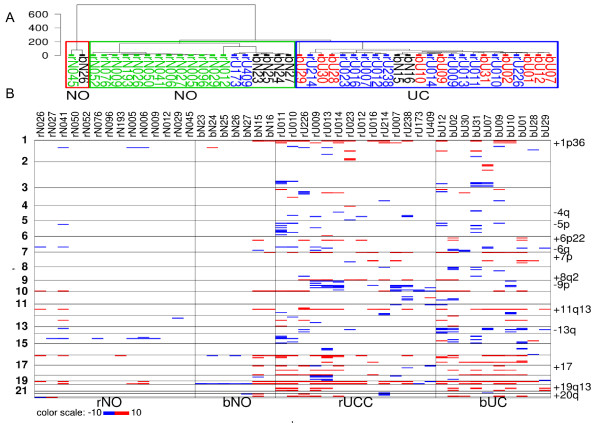
**The summarization of gene expressions grouped on physical locations of genomic cytobands, relative to normal samples from bladder**. bNO: normal samples from bladder; rNO: normal samples from renal pelvis; bUC: bladder UC; rUC: renal pelvis UC. **A) **Unsupervised clustering result of the samples using raw summarization data. The majority of tumor and normal samples are separated, while the rUCs and bUCs are mixed with each other, showing indistinguishable summarization profiles. **B) **The summarization plot reflects underlying genomic imbalances in our study population and correctly identifies the most frequently observed cytogenetic alterations previously documented in UC (marked on right margin of the plot).

Chromosomal defects such as chromosome amplifications and deletions can be detected from the analysis of gene expression data [22,23]. For example, genes that map to regions of chromosome amplification typically produce more RNA than is found in cytogenetically normal samples; likewise, genes that map to regions of chromosome deletion typically produce less RNA. We scanned the gene expression data to determine if UCs isolated from the renal pelvis showed a different spectrum of chromosomal abnormalities than UCs isolated from the bladder. Figure 1A shows the unsupervised clustering results, while Figure 1B shows a summary of potential chromosomal abnormalities based on gene expression data as described in the Materials and Methods. Briefly, expression values were grouped into sets based on gene location (to the nearest cytoband) and the expression of genes in the tumor sample were compared to the expression in the normal bladder samples (bUC). While Figure 1A was produced on the summarization data set without cutoff values, Figure 1B was produced by using cutoff values of 4.3 from above and -4.3 from below on the summarization scores (any value in between was set to 0). Cytobands with fewer than 10 genes in our database were removed, as too few genes in a cytoband often produce undesired summarization results.

This figure serves as an overview of the data set and a summary of deviation (bias) of gene expressions from normal references values, as well as a tool for inferring the cytogenetic alterations underlying the gene expression profiles (see Materials and Methods). As anticipated, benign urothelial samples from the bladder and renal pelvis did not show many large-scale gene expression alterations. At cytogenetic level, we did not detect many differences between rUC and bUC, as seen in Figure 1A, where rUC and bUC samples were well mixed with each other. The exception was an apparent increase in the overall 9p loss in rUC, which is the region containing the cyclin-dependent kinase inhibitor 2A and 2B (*CDKN2A *and *CDKN2B*) genes. Both *CDKN2A *and *CDKN2B *losses have been well documented in UC, with a *CDKN2A *deletion often associated with worsened outcomes [24,25]. Losses in these genes have also been implicated in other RCC subtypes [26]. This method predicted correctly the following cytogenetic alterations that are frequently observed in UCs: +1p36, -4q, -5p, +6p22, -6q, +7p, +8q2, -9p, +11q13, -13q, +17, +19q13, and +20q [27-29]. While this surrogate for detecting genomic gains or losses is approximate and has limitations, the pattern of chromosomal abnormalities detected does not indicate a gross difference in aneuploidy between the renal and bladder-derived UCs [30].

In our subsequent data analyses (Figure 1B), we noticed that two bUCs (bU28 and bU29) and two rUCs (rU173 and rU409) displayed patterns distinct from those of other samples in their respective classes. Their gene expression summarization profiles appeared more like the profiles from normal samples than from tumors. When they were included in the supervised or unsupervised clustering study, these four samples always segregated within the same cluster as the normal samples. These observations lead us to believe that these four samples may be inherently different from the rest of the tumor samples or that their mRNA quality may have been poor.

Indeed, a careful retrospective review of the samples revealed that bU28 had approximately 50% stroma admixed, while bU29 had a lesser (but still significant) amount. This admixture likely affected the gene expression measurement. Although the urothelium in normal samples was stripped, a small portion of the underlying lamina propria/connective tissue remains inherently attached to the base of the specimen and may explain the clustering of these tumor specimens with normal urothelium. On the other hand, both rU409 and rU173 represent high-grade invasive UC, with rU409 having a prominent papillary component. Overall, it is unclear why they have gene expression profiles so close to that of normal urothelium. Tissue sample bias for running the microarray may be one of the possible reasons. In our subsequent analyses, these four samples have been removed from study to avoid skewing the results. A summarization box plot of the data set was shown in Additional File 1: Supplemental Figure S1. Apart from rNO samples, which appeared to have narrower IQRs, all the rest of the samples had comparable data summarizations. We are not sure if this is due to the batch effect or if this reflected the real differences between rNO and other samples. Tissues for rNO cases were obtained from the same institute as those for rUCs; their microarray data were scanned using the same facility and in roughly the same time period as data for rUC samples. In both cases, the difference between rNO and the rest of the samples will have no effect on our conclusions here, as our main purpose was to compare the profiles between rUC and bUC. In general, our microarray data set was of good quality for profiling purposes.

### Unsupervised clustering reveals similar gene expression profiles in UC but not in normal urothelium

**Figure 2:**
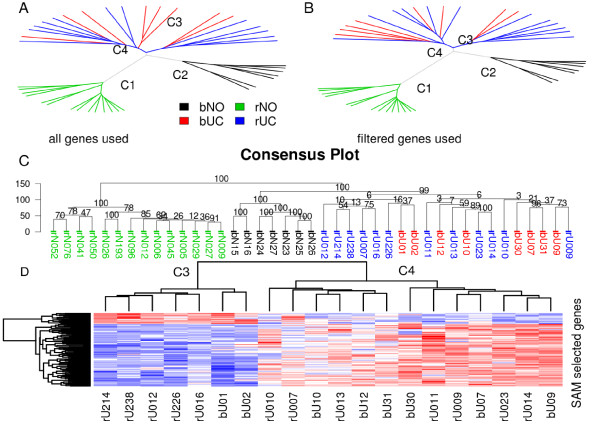
**Unsupervised clustering analysis**. **A) **Phylogenetic plot of the samples using all genes in the database (17,589 in total). **B) **Similar plot based on 3518 genes filtered by interquartile range (IQR > 0.9) and coefficient of variation (CV > 0.1) to remove genes with small intensity variations among the studied samples. **C) **Consensus plot based on the filtered genes; 500 clustering trees were generated by bootstrapped genes. The numbers on the branching lines are the percentage of times that the specific cluster occurred among the 500 trees. Normal samples from either renal pelvis or bladder always clustered with their respective groups (100%), whereas upper- and lower-tract UCs demonstrated frequent overlap. **D) **Heat map of SAM-selected genes that are the most differentiated between UC samples in the C3 and C4 groups identified in the unsupervised study (Figure 2A and B).

To compare the gene expression profiles of all samples, we first performed an unsupervised clustering study using all genes in the database (>17,000; Figure 2A). Several key findings emerged from this primary analysis. First, normal samples from the renal pelvis were tightly clustered together far from the remaining samples (cluster C1 in Figure 2A), indicating they have a highly distinct gene expression profile relative to either normal bladder urothelium or UC. Second, normal bladder urothelium also clustered together tightly (cluster C2 in Figure 2A). These findings indicate that at the genetic level, normal urothelium can be robustly distinguished from UC, and that normal urothelium from the upper and lower tracts have unique profiles despite identical morphological appearance.

We next compared the relationship between rUC and bUC. In this unsupervised clustering analysis, rUC and bUC had overlapping profile patterns, suggesting that the UCs from the two locations are indistinguishable at the genome-wide gene expression level, although two distinct groupings appeared to emerge within the total tumor group. This was also true when the unsupervised clustering was applied without normal renal samples (data not shown) to exclude the possibility of renal parenchyma contamination.

To test whether the clustering patterns depended on the number of genes evaluated, we next applied various filtering steps to decrease the number of genes before applying the clustering algorithm. One choice was to use IQR and CV as our filtering criteria. About 3500 genes passed these filtering criteria (see Materials and Methods). The clustering result is displayed in Figure 2B and is consistent with the unfiltered data. Next, we applied the consensus algorithm to the filtered gene set to determine the stability of the clusters (Figure 2C). Five hundred trees were built and the frequencies of clusters were counted among all 500 trees. The frequencies are indicated in the nodes of the tree plot in Figure 2C. We found that the normal samples from renal pelvis and the normal samples from bladder always clustered together within their respective groups, distinguishable from the rest of samples (100% in both cases). The tumor samples in total also consistently clustered together more than 99.9% of the time (the numbers in Figure 2C were truncated to whole numbers), although two overlapping groups (C3 and C4), each containing both rUC and bUC samples, were identified. To identify the underlying gene expression profiles, we used the SAM method to select the most differentially expressed genes between samples of the two clusters (C3 and C4). The heat map of the selected 95 genes (Figure 2D, Additional File 2: Supplementary Table S1) shows that these genes clearly separate the samples into two classes, as was expected.

The reason for this dual clustering pattern of UCs (C3 and C4) is currently unclear, but it was not the result of confounding effects (day the arrays were performed, lot number of chip, quality of RNA, etc.). We suspect that the pattern may be inherently related to differences in the underlying tumor biology and/or behavior; future studies will address this possibility in more detail. Overall, the unsupervised clustering of gene profiles revealed that it is readily possible to distinguish normal urothelium from UC, as well as to distinguish normal urothelium from the upper and lower tract, but it cannot be used to determine whether a UC is derived from the upper or lower urinary tract.

### Supervised study of UCs reveals marked commonality in gene profiles

**Figure 3:**
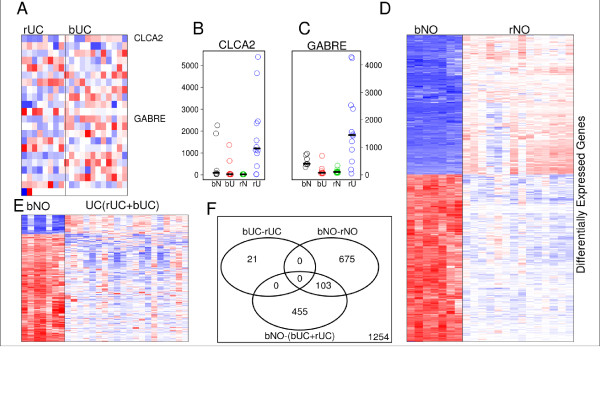
**Supervised analysis studies to identify genes differentially expressed**. **A) **Twenty-one genes that best distinguish UC of the renal pelvis and UC of bladder. q-values were obtained by the FDR controlling procedure on the p-values with Wilcoxon rank sum and signed rank tests. **B-C**) Gene expression profiles of the genes *CLCA2 *and *GABRA*, showing significantly overexpression in bUC relative to rUC (potential biomarkers for distinguishing between bUC and rUC). Bars show the median values for each class. **D) **Normal samples from bladder and renal pelvis, 778 genes identified, see Additional File 4: Supplementary Table S2, and **E) **normal bladder and combined UC samples (bUC+rUC as a single class), 558 genes. See Additional File 5: Supplementary Table S3. **F) **Venn diagram of gene lists in A, D, E).

We next compared the samples directly to obtain the most informative genes between UCs of either origin using the statistical analysis of microarray (SAM) approach [18]. The resulting genes are generally regarded to be the "best" for separating the two classes of samples (most varied among the samples, plus having the most differential ability between the two classes). The best 21 genes are listed in Table 2 (by setting the delta value equal to 0.37 in the SAM algorithm; it was liberal in order to have some genes left), and the heat map of these genes is shown in Figure 3A. All q-values were insignificant, with the exception of that for the *CLYBL*-gene, which showed a marginally significant q-value 0.06 at FDR level 0.05. By systematically checking the gene expression profiles individually for each gene in the list, we found that no single gene showed expression intensities uniformly higher or lower in bUC versus rUC. The absence of genes that significantly differentiate between rUC and bUC further supports the overall related nature of these two entities.

**Table 2:** The 21 most informative genes identified by the SAM method between bladder UCs and UCs from renal pelvis.

Entrez Gene ID	Symbol	FC^1^	p^2^	q^3^
171425	*CLYBL*	-2.47	1.60E-05	0.0555

2564	*GABRE*	-9.65	0.003	0.462

10863	*ADAM28*	-2.73	0.00048	0.357

642587	*LOC642587*	-3.75	0.0022	0.419

6414	*SEPP1*	-3.9	0.0022	0.419

84674	*CARD6*	-2.44	0.0022	0.419

2770	*GNAI1*	-3.07	0.0073	0.501

27303	*RBMS3*	-2.56	0.0015	0.419

9635	*CLCA2*	-11.9	0.012	0.549

10158	*PDZK1IP1*	-4.68	0.0041	0.462

143686	*SESN3*	-2.91	0.0041	0.462

9976	*CLEC2B*	-2.71	0.0055	0.48

51299	*NRN1*	-5.27	0.0055	0.48

4150	*MAZ*	2.74	0.00019	0.333

55388	*MCM10*	2.15	0.0015	0.419

1104	*RCC1*	1.67	0.00071	0.357

1116	*CHI3L1*	7.36	0.0041	0.462

699	*BUB1*	2.2	0.0041	0.462

345557	*PLCXD3*	3.63	0.047	0.633

79827	*ASAM*	2.61	0.0022	0.419

1503	*CTPS*	1.48	0.00071	0.357

^1^Fold change of bUC relative to rUC.

^2^*p *values were based on Wilcoxon rank sum and signed rank tests.

^3^q values were calculated by Storey's procedure.

Based on the profiles of the genes in the supervised study, the most promising genes to differentiate the tumors are chloride channel, calcium-activated, 2 (*CLCA2*, FC = 12, Figure 3B) and gamma-aminobutyric acid (GABA) A receptor, epsilon (*GABRE*, FC = 9.6, Figure 3C). Both genes are frequently overexpressed in rUC relative to both bUC and to primary renal carcinomas (Additional File 3: Supplemental Figure S2A, S2B). These two genes are actually related to the same functional modules. Through queries to a public database, we found that both genes share the same biological function of catalyzing and facilitating diffusion of chloride through a transmembrane aqueous pore or channel without evidence for a carrier-mediated mechanism (gene ontology GO:0005254,) and chloride ion binding (GO:0031404,). We recently showed that FXYD domain containing ion transport regulator 3 (*FXYD3*), another gene related to these functional modules, was identified as a marker gene for UCs from the renal pelvis (Zhang et al, 2009, in review). Of note, *CLCA2 *represents a tumor suppressor gene that has been shown to promote apoptosis in a variety of cancer models, including breast cancer [31,32]. Its role in UC is currently unclear. Future studies to delineate the role of these chloride-mediating factors in upper- and lower-tract UC may reveal distinct biological functions of these molecules, as well as provide a potential diagnostic tool for use with urine specimens.

### Supervised studies of gene expression profiles between other pairs of classes

Using same method of identifying differential genes as above, we next compared the gene expression profiles between normal samples from the bladder and normal samples from the renal pelvis (Figure 3D), and between normal samples from the bladder and combined UC samples (bUC and rUC, Figure 3E). With similar parameters for the SAM algorithm, there are 558 genes significantly differentiated between bNO and UC and 778 genes between bNO and rNO. The large number of differentially expressed genes in the latter comparison implies the large number of profile dissimilarities between bNO and rNO, as was seen in Figure 2A-2C, clusters C1 and C2. Unlike the comparison between bUC and rUC (Figure 3A), results from these two pairs of comparisons show remarkable differences in gene expression profiles, largely in agreement with our unsupervised study results in Figure 2. The gene lists are given in detail as supplementary material (see Additional Files 4 and 5: Supplementary Table S2 and S3).

While about half of the genes in the list are overexpressed in rNO relative to bNO, the majority of genes identified to be significant between UC and normal samples are underexpressed in UC relative to bNO. Among the genes differentially expressed between bNO and rNO, a significant number are sodium-related (15 genes in total), involving functions related to sodium transportation (sodium ion binding, sodium ion transportation). Examples of genes include ATPase Na+/K+ transporting beta 1 (*ATP1B1*), FXYD domain ion transport regulator 2-5 (*FXYD2-5*), sodium channel, non-voltage-gated 1 alpha and gamma (*SCNN1A, SCNN1G*), and several genes in solute carrier family.

Interestingly, several genes involving angiogenesis or vasculature development showed decreased gene expression in UC samples relative to bNO samples, such as endothelial differentiation, sphingolipid G-protein-coupled receptor, 1 *(EDG1)*, transcription factor 21 *(TCF21*), apolipoprotein L domain containing 1 *(APOLD1)*, endomucin *(EMCN)*, forkhead box F1 *(FOXF1)*, and reversion-inducing-cysteine-rich protein with kazal motifs *(RECK)*.

## Discussion

In this study, we have compared the gene expression profiles of urothelial carcinomas arising from the upper and lower urinary tract, as well as their relationship to profiles from normal urothelium at both locations. Our unsupervised clustering analyses showed that UCs from the two locations often showed overlapping gene expression profiles, despite their different embryonic origins. Our supervised clustering analysis showed the existence of very limited differences in gene expression profiles and in signaling pathways between UCs from the two locations, although *CLCA2 *and *GABRE *may hold promise as differential markers.

Of interest, we identified a distinct genetic profile between normal urothelium derived from the upper and lower tract. Although identical in morphological appearance, the urothelium of the lower tract is derived from the upper portion of the urogenital sinus, while that of the upper tract is from the ureteric bud of the mesonephric duct. The former is potentially exposed to a higher concentration of excreted toxins due to the storage function of the bladder. This may explain the differences in gene expression profiles of the normal samples from the two locations. Paradoxically, the striking gene expression profile differences between bNO and rNO did not imply the differences in regional gene expression bias (see Figure 1). This implies that the profile differences between bNO and rNO are not a result of underlying genetic differences. We retrospectively evaluated the genetic background (race, gender, etc.) of individuals whose specimens were used for this study and identified no significant differences between the sample populations (data not shown).

Based on the differences in profiles between rNO and bNO, we have used benign bladder urothelium as the general reference point in producing the gene expression summarization plot in our data analysis. Our result confirms that the genetic backgrounds of UCs from both locations are largely indistinguishable, offering support for the idea that they are largely the same tumors, sharing the same genetic abnormalities. Gene therapy treatments that are effective against one of them are quite likely to be effective against the other due to their similar genetic backgrounds.

Our study shows that the phenotypic differences between rUC and bUC are likely to have non-molecular origins. For example, one such difference is the survival prognosis. It has been demonstrated that patients with rUC have significantly poorer survival than patients with bUC [1]. However, these phenotypic differences do not necessarily imply genetic differences. Studies show that UCs from renal pelvis are more frequently at a late stage when diagnosed than UCs from bladder [2-5]. The anatomic and environmental surroundings of the tumor cells may also play a role in the phenotypic differences.

Our study shows that if there are any genetic contributions toward the phenotypic differences, that contribution is very limited, possibly restricted to only one signaling pathway. Our direct comparison did not show strong evidence that there were genes significantly differentiated between tumors from the two locations (see Figure 3A). Even if a study finds a difference in gene expression between upper and lower GU tract tumors, this still does not necessarily imply that the difference is accounted for by the aggressiveness of the upper-tract tumors. For example, in a case where the elevated genes are mostly stromal genes, the elevation in gene expressions can be easily explained by the presence of more benign stromal tissues in the bladder samples than in the upper-tract samples. Similarly, genes related to ion metabolism could be found elevated in the upper-tract tumors because of the presence of benign renal parenchymal tissue in those samples.

Because the gene expression profiles are very similar between UCs from the upper and lower tracts, it is unlikely that direct cytogenetic profile comparisons will find any significant differences. Indeed, our analysis of regional gene expression bias identified correctly that the most common cytogenetic alterations seem to appear in both rUCs and bUCs. These cytogenetic alterations include +1p36, +6p22, +7, +8q22, -9p21, +11q, -13q, +17, +19q13, and +20q, which have all been previously documented in UC [27-29]. Due to the indirect method, the complex gene regulations and gene interactions (including unknown mechanisms for example, in [30]), as well as noise within the data, the results may not agree with results obtained from direct measurement of sample DNA materials (such as from SNP arrays).

In summary, we compared the gene expression profiles of UCs arising from the upper and lower urinary tract, as well as the profiles of normal urothelium from the renal pelvis and bladder. We did not identify significant differences between upper- and lower-tract UCs, suggesting that the entities are closely related and therefore will share molecular features that can be maximized in the development of new diagnostic markers and therapeutic interventions. Further evaluation of potential differences in the chloride transporter pathway will be conducted to determine whether *CLCA2 *and *GABRE *may reflect unique markers that can be used to differentially evaluate and target upper-tract UC.

## Conclusions

In summary, we found that the gene expression profiles of UCs from the upper and lower tract were extremely similar, suggesting that similar pathogenic mechanisms likely function in the development of these tumors. While there may be small differences between these profiles, they are likely to be restricted to a very limited number of gene pathways. Therefore, it may be possible to treat the UCs from both locations as a single entity on a genetic level in the future.

## Competing interests

The authors declare that they have no competing interests.

## Authors' contributions

ZZ analyzed the data and wrote the manuscript. XJY provided pathologic expertise. BTT and KAF participated in the design and coordination of the study and helped to write the manuscript. DEH contributed and evaluated the samples and helped to write the manuscript. All authors read and approved the final manuscript.

## Pre-publication history

The pre-publication history for this paper can be accessed here:

http://www.biomedcentral.com/1755-8794/3/58/prepub

## Supplementary Material

Additional file 1 **Supplementary Figure S1**. Box plot of the gene expression intensities of the samples, using intensities of all genes in the database on a logarithmic scale after normalization. Click here for file

Additional file 2 **Supplementary Table S1**. Lists of genes differentially expressed between C3 and C4 classes identified in Figure 2A-C by SAM method using the default cutoff value 0.05 on the adjusted p-values. Click here for file

Additional file 3 **Supplementary Figure S2**. Gene expression profiles of *CLCA2 *(A) and *GABRE *(B) in normal urothelium, UC, and subtypes of renal cell carcinoma. Click here for file

Additional file 4 **Supplementary Table S2**. Lists of genes differentially expressed between rNO and bNO identified in Figure 2D by SAM method using cutoff value 0.001 on the adjusted p-values. Click here for file

Additional file 5 **Supplementary Table S3**. Lists of genes differentially expressed between bNO and UC (bUC and rUC) identified in Figure 2E by SAM method using cutoff value 0.001 on the adjusted p-values. Click here for file
