# Supplementary material for: Comparative gene expression profiling analysis of urothelial carcinoma of the renal pelvis and bladder
Source: BMC Med Genomics. 2010 Dec 15;3:58. doi: 10.1186/1755-8794-3-58 (PMC3022544; doi:10.1186/1755-8794-3-58)
Supplement: Additional file 1 — Supplementary Figure S1. Box plot of the gene expression intensities of the samples, using intensities of all genes in the database on a logarithmic scale after normalization. [file 1755-8794-3-58-S1.PDF]

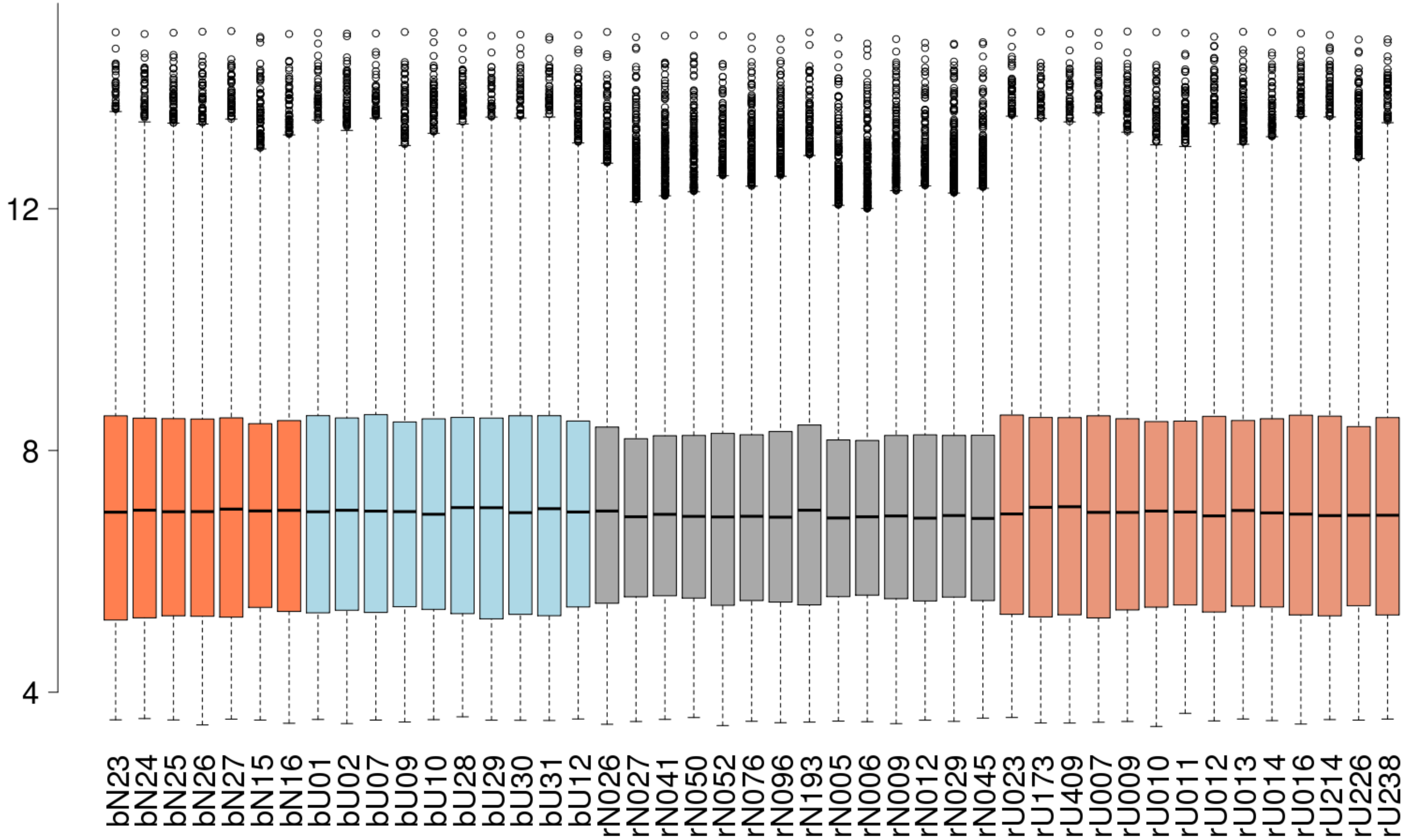

12
8
4
bN23
bN24
bN25
bN26
bN27
bN15
bN16
bU01
bU02
bU07
bU09
bU10
bU28
bU29
bU30
bU31
bU12
rN026
rN027
rN041
rN050
rN052
rN076
rN096
rN193
rN005
rN006
rN009
rN012
rN029
rN045
rU023
rU173
rU409
rU007
rU009
rU010
rU011
rU012
rU013
rU014
rU016
rU214
rU226
rU238
