# Supplementary material for: Comparative gene expression profiling analysis of urothelial carcinoma of the renal pelvis and bladder
Source: BMC Med Genomics. 2010 Dec 15;3:58. doi: 10.1186/1755-8794-3-58 (PMC3022544; doi:10.1186/1755-8794-3-58)
Supplement: Additional file 3 — Supplementary Figure S2. Gene expression profiles of CLCA2 (A) and GABRE (B) in normal urothelium, UC, and subtypes of renal cell carcinoma. [file 1755-8794-3-58-S3.PDF]

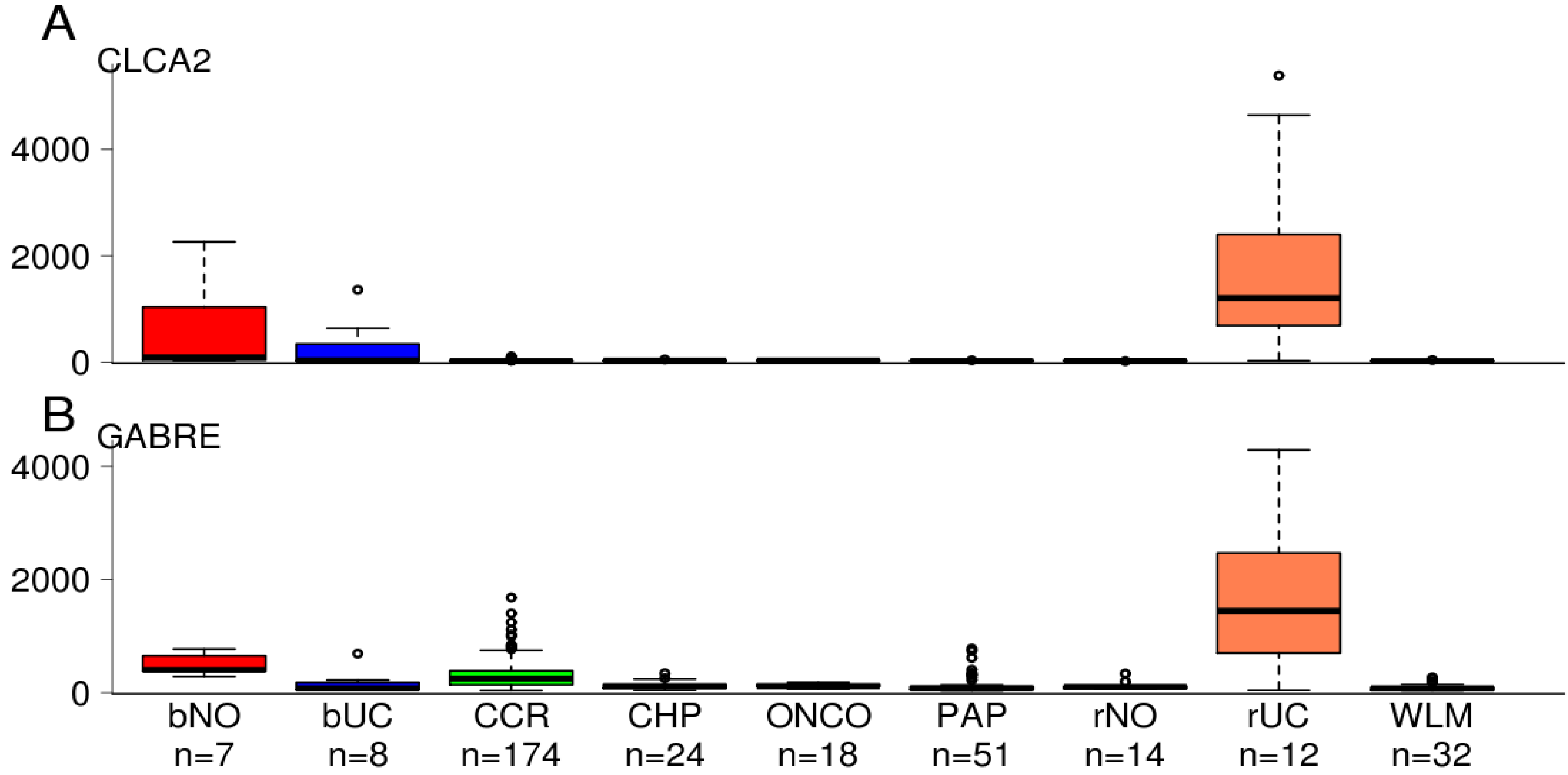

Notations: CCR: clear cell renal carcinoma, CHP: chromophobe renal carcinoma, ONCO: oncocytoma, PAP: papillary renal carcinoma, WLM: wilm tumor, bUC, rUC: urothelial carcinoma from bladder or renal pelvis. bNO, rNO: benign samples.
